# Potential hazard characteristics of trees with hollows, cavities and fruiting bodies growing along pedestrian routes

**DOI:** 10.1038/s41598-022-25946-0

**Published:** 2022-12-10

**Authors:** Marzena Suchocka, Magdalena Wojnowska-Heciak, Paweł Jankowski, Jacek Mojski, Agata Milanowska, Marcin Kubus, Hazem M. Kalaji

**Affiliations:** 1 Department of Landscape Architecture, Institute of Environmental Engineering, Warsaw University of Life Sciences - SGGW, Nowoursynowska St. 159, 02-776 Warsaw, Poland; 2 Department of Computer Information Systems, Institute of Information Technology, Warsaw University of Life Sciences - SGGW, Nowoursynowska St. 166, 02-776 Warsaw, Poland; 3 Fundacja Zielona Infrastruktura, Wiatraki St. 3E, 21-400 Lukow, Poland; 4 Twój Świat, Ul. Okrzei 39, 21-400 Łuków, Poland; 5 Department of Landscape Architecture, West Pomeranian Univeristy of Technology, Papieża Pawła VI St. 3a, 71-459 Szczecin, Poland; 6 Institute of Technology and Life Sciences - National Research Institute, Al. Hrabska 3, 05-090 Falenty, Raszyn, Poland; 7 Department of Plant Physiology, Institute of Biology, Warsaw, University of Life Sciences - SGGW, Warsaw, Poland

**Keywords:** Biodiversity, Climate-change ecology, Urban ecology

## Abstract

This article is a study of risk assessment of trees with hollows, cavities and fruiting bodies for the improvement of the management and protection of urban trees growing along pedestrian routes. 317 trees were examined using TRAQ risk classes, VTA and ISA BMP methodology, Roloff's vitality classification, and sonic tomography (SoT) during the spring and summer of 2021. The collected data was analysed using the Kruskal–Wallis H-test, the Dunn multiple comparison test, the pairwise comparison of proportions with Holm correction, the U-Manna-Whitney test, and the Fisher exact test. The analysed trees grow alongside public footpaths and footways in central Zakopane, Poland. The study results indicate that tree trunk hollows are judged to have no adverse effects on a tree’s vitality when assessed using visual methods and are deemed to have a limited effect on vitality estimated with SoT. Though most high and moderate-risk trees, according to SoT (88% and 80%, respectively), had hollows, such trees were a small fraction of all 171 trees with hollows, cavities and/or fruiting bodies, 2.3% and 8.8%, respectively. Therefore, the decision to remove a tree should be based on advice from a professional arborist, supported by sonic tomography (SoT) or similar objective methods.

## Introduction

Street trees are an essential element in urban contexts^1,2^. Limited space for planting affects tree vitality^3^. Trees planted along pedestrian paths are the most accessible means for urban residents to access nature^4,5^. Urban trees provide various tree-based ecosystem services, ranging from helping to mitigate climate change and protecting biodiversity to mitigating pollution and noise reduction^6,7^, as well as ensuring residents' overall well-being, especially in crises^5,8^. As a tree ages, it plays an increasingly important role in biodiversity conservation, including trees with cavities or hollows termed ‘hollow bearing trees’ or ‘habitat trees’^9,10^. In addition to providing significant benefits to birds^11^, mammals (e.g., red squirrels), mosses, lichens, fungi^12^, saproxylic insects^13^, and bats in marginal areas,^14^, old trees ensure ecosystem diversity in hard-to-reach regions^15,16^.

The habitat in which a tree grows affects its longevity. In areas with limited planting space and anthropogenic pressures (soil compaction, salinity, mechanical damage), urban street trees may exhibit advanced life stage characteristics earlier than trees growing in urban parks or urban forests^17^. Challenging habitat conditions are also a reason for signs of premature ageing in trees (including hollows and cavities). Trunk decay, in the form of a cavity, is a typical feature of old trees^18,19^, but few street trees reach this age^20,21^. As trees which line streets are exposed to more extreme conditions than trees located along sidewalks in urban parks^22,23^, there is an increased risk of them developing cavities due to their sensitive location.

Locations along pedestrian paths with varying degrees of tree cover and occupancy^6^ reduce the level of safety perceived by urban residents^24^. Failure potential applies, at some level, to all trees^25,26^ and depends on various factors, including tree health^27^, presence of decay^28–32^, and maintenance histor^33–35^. Improper maintenance, such as heavy pruning, may result in not only a diminished tree crown capacity but also exposure to fungal infection, dieback occurrence and a higher risk of failure in mature trees^35–38^.

As landowners, including local authorities, are responsible for the safety of users, road managers face the problem of risk management in particularly vulnerable roadside zones^39,40^. Significant problems associated with urban tree maintenance and management cover residents’ safety and repair costs related to damaged infrastructure, assets, or risks to human life and health caused by street trees^41–43^. Compensation can be a significant percentage of the annual tree maintenance expenditure^41^. In order to prevent breakage due to strength failure or damage caused by decay, municipalities use various techniques to detect structural defects in trees^40,43^.

Tree risk assessment supports the process of correct urban tree management. Tree risk assessment covers identifying, analysing and evaluating the risk of (1) failure potential (the likelihood that all or part of the tree will fail), (2) likelihood of impact (the likelihood of an object or person being present/ struck), (3) the consequences of failure (i.e., personal injury, damage to property, or disruption of services/activities)^44–47^. An experienced arborist often uses visual assessment methods as reliable evidence of tree hazard potential^8,48,49^. The most commonly used risk tree assessment methods are: ISA Tree Risk Assessment Qualification (TRAQ)^50^, ISA/M&C^51^, ISA/BMP^46,51^, Quantified Tree Risk Assessment (QTRA)^52^; “Tree Hazard: Risk Evaluation and Treatment System” (THREATS)^53^; Visual Tree Assessment (VTA)^44^, “A Guide to Identifying, Assessing, and Managing Hazard Trees in Developed Recreational Sites of the Northern Rocky Mountains and the Intermountain West” and “Guide to Hazard Tree Management” promoted by USDA^54,55^, differ in terms of how they weight each underlying risk factor and tree defects; and how the various components are combined into a final risk determination^51,57^. The training available to arborists influences the assessment method chosen, and while TRAQ is commonly applied in North America^58^, QTRA is used in the United Kingdom, Australia, and New Zealand^56,60^.

However, these methods often fail to convince private and governmental decision-makers of low tree failure, especially when trunks have a hollow^16^. Three commonly used advanced technical tests support the tree removal decision process: Resistance recording drill^37,61^ and sonic tomography (SoT)^57,58^ are used to evaluate stem rot and a pulling test^59,60^ to test to analyse the root system and quantitative measurements of tree risk assessment (i.e., target occupancy of the site, the size of a tree or a tree part). However, the decision on tree health is dependent on the assessor’s judgment^25,52^. There is evidence to suggest that depending on the tree assessor’s background (i.e., whether they have children^61^, whether the tree poses a direct threat to pedestrians, or whether the assessor has completed specialised training on tree health measurement and holds industry credentials)^62^ the risk perception may differ. The risk perceived by the assessor may not correlate with tree health^45,63^. People’s subjective perceptions often play a decisive role in deciding whether to remove a tree with visible signs of cavities and/or fungal infestation rather than scientific evidence or expert recommendations^7^.

Hollows, cavities, and fungi on trunks or branches often raise concerns about potential safety risks to the public and infrastructure (falling branches; trees uprooted or toppled by wind)^64–66^. In addition, the decay of standing trees is a significant safety concern^67^, as the trunk, branches, or roots can be weakened, increasing the risk of structural failure. Therefore, hollow trees are particularly vulnerable to removal in urban landscapes^68,69^. In terms of aesthetics, trees with ageing characteristics are generally accepted^7,70^. However, municipal preventive measures for pedestrian and vehicle safety often mean old trees are replaced with younger ones, especially along pavements^71^.

However, there is evidence that even a safe tree can be almost entirely hollow^72^. A larger hollow is not an automatic reason for felling. The branch breakage risk is similar for hollow trees to those without hollows.^72^. The development of decay depends on the influence of three factors that affect the interaction between the host (tree) and the decay fungus: the spore load of the fungus, the environment (microbial growth conditions), and the susceptibility of the tissues, which are influenced by the vitality of the tree, among other factors^73^. Appropriate management decisions must distinguish between dangerous rot and rot with no safety implications, especially for valuable urban trees.

Although several researchers have published results on material properties (e.g., compressive strength and Young's modulus), there is very little analysis to verify the use of SoT on hollow trees in urban areas. It is important to know whether hollow trees planted along footpaths through parks and footways along streets are as dangerous as their perceived risk and whether the presence of cavities and/or fungi that cause fears related to potential safety risks should be considered as reasons for tree removal decisions. Therefore, the main objective of this study is to increase knowledge about the risk posed by hollow street trees. In addition, the results will fill a research gap on the presence of hollow trees in urban contexts located along footpaths through parks and footways along streets as places of possible periods of high occupancy.

## Methods

### Case study setting

The research project presented here was established to improve the management and protection of urban trees with hollows or fruiting fungi in Zakopane, Poland. It was conducted to assist authorities in removing potentially hazardous trees located along main footpaths in selected parks and along selected footways by main streets. The 2021 tree inspection consisted of a visual tree assessment supported by a sonic tomographic survey of Zakopane's most common urban tree species. The risk assessment was conducted as a case study. The Municipality of Zakopane provided funding for the study as part of the regular tree monitoring and assessment. Therefore, the scope of the study (footpaths through parks and footways along streets) was determined at the request of the Zakopane Municipality.

### Tree sample

The trees selected for the study were among the highest-risk trees: trees growing along footpaths through two parks and footways along selected main streets in the centre of Zakopane, Poland. The most frequently used streets and park paths aligned with trees were selected for the study. As the research concerned urban street trees, the target (likelihood of impact) has been determined as constant. The occupancy rate, target zone in relation to the tree or tree part and consequences, considering target, part size, fall distance, and target protection, was the basis to determine the risk of harm.

**Figure 1:**
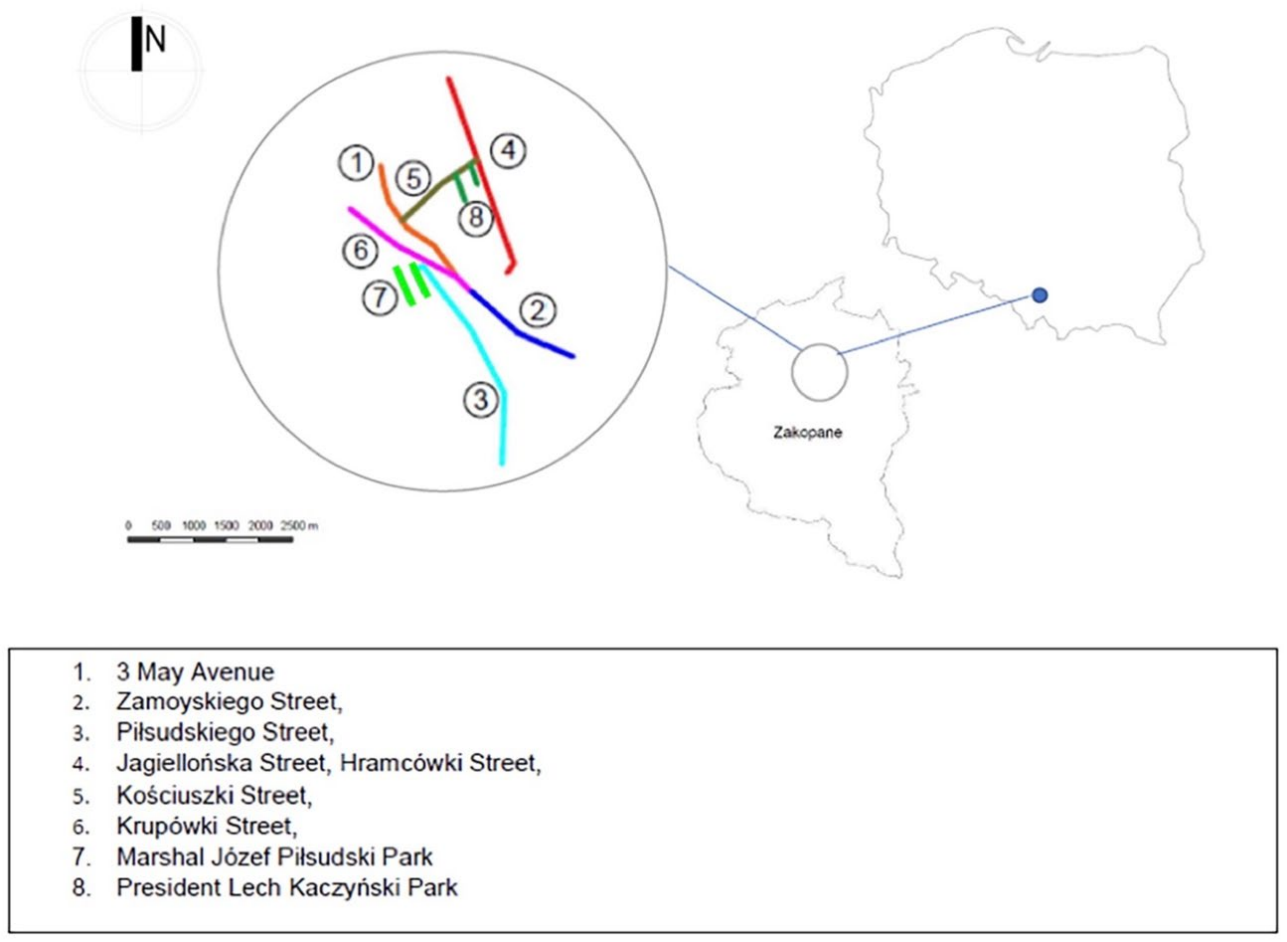
Location of all the studied trees (*Source*: Own elaboration).

**Figure 2:**
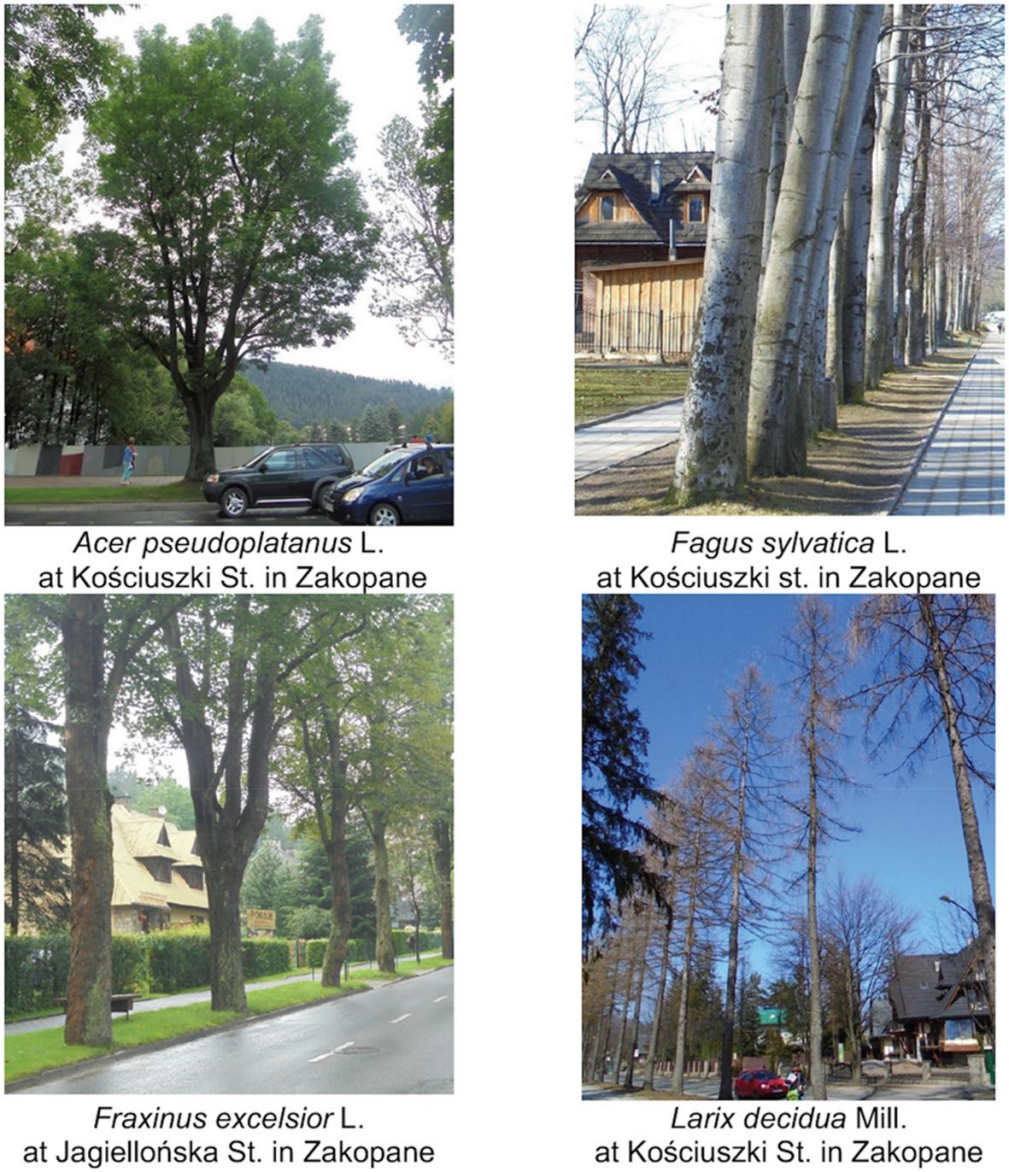
Views of the studied trees (*Source*: Marzena Suchocka).

The exact location of the studied trees can be seen in Fig. 1. The chosen examples of studied trees are illustrated in Fig. 2. Data was collected in the spring and summer of 2021. The database contained 326 trees. Of these trees, 317 individuals were selected for a complete analysis. The sample included 23 tree species, 234 trees growing along footways along the streets, 83 along footpaths, and 171 with visible hollows.

The term “trees growing along the footways” covers trees located along the pavements next to roads^74^. In these locations, there is greater anthropopression from the vehicular traffic on the trees' root system. The term “trees growing along footpaths” covers only trees located along the paths in parks^74^. A detailed list of tree species included in the study is shown in Table 1.

**Table 1:** Information about trees examined (source: Processed by the Authors).

No	*Species*		Location		Hollow/cavity/fungus occurrence	
			Along the footpaths (in parks)	Along the footways (by streets)	No	Yes
1	*Abies concolor*	1	–	1	–	1
2	*Acer_platanoides*	3	1	2	1	2
**3**	***Acer pseudoplatanus***	**45**	**11**	**34**	**32**	**13**
4	*Acer_saccharinum*	2	1	1	1	1
5	*Acer_saccharum*	1	1	–	–	1
6	*Aesculus_hippocastanum*	3	–	3	–	3
7	*Betula_pendula*	3	–	3	1	2
**8**	***Fagus_sylvatica***	**73**	**49**	**24**	**19**	**54**
**9**	***Fraxinus_excelsior***	**120**	**10**	**110**	**66**	**54**
**10**	***Larix_decidua***	**24**	**1**	**23**	**14**	**10**
11	*Phellodendron_amurense*	1	1	–	–	1
12	*Picea_pungens*_f*_glaca*	2	–	2	1	1
13	*Pinus_nigra*	3	1	2	2	1
14	*Pinus_strobus*	1	–	1	–	1
15	*Populus__* × *canadensis* ‘Marilandica’	1	–	1	1	–
16	*Populus_*sp.	1	–	1	–	1
17	*Prunus_avium*	1	–	1	–	1
18	*Quercus_*sp*.*	2	–	2	–	2
19	*Salix_fragilis*	1	–	1	–	1
20	*Sorbus_aucuparia*	11	–	11	2	9
21	*Tilia_cordata*	13	5	8	5	8
22	*Tilia_platyphyllos*	4	2	2	–	4
23	*Ulmus_glabra*	1	–	1	1	–
	TOTAL	317	83	234	146	171

In bold: tree species with total samples exceeding 20, chosen for detailed analysis.

### Research involving plants

Our research covered field study observations and non-invasive measurements of trees without collecting plant material. Therefore, in compliancewith the IUCN Policy Statement on Research Involving Species at Risk of Extinction and the Convention on the Trade in Endangered Species of Wild Fauna and Flora, no permissions were needed.

### Tree vitality assessment

The tree assessment was conducted in cooperation with the city as part of the city's tree maintenance activities. Each tree was evaluated according to Roloff's (2015) visual classification and based on the vibrancy of the distal portions of the crown^75^. Roloff’s classification, as a visual assessment of tree vitality, was used when assessing the above-ground crown structure (visible and physiological state of branch architecture). Roloff’s classification aims to determine/assess tree vigour and vitality using leaves, i.e., crown transparency, by branching pattern^75^. Trees were categorised into four groups: R0' Exploration' Trees in the phase of intense shoot growth, R1' Degeneration': Trees with slightly delayed shoot growth, R2' Stagnation': Trees with visibly delayed shoot growth, R3' Resignation': Trees with no possibility of regeneration and no return to the second class.

Taking into consideration the availability and suitability of the study’s needs^76^, the selected methods included a combination of VTA, ISA/BMP and TRAQ^77,78^. We decided first to use VTA and ISA/BMP approach and to look for noticeable defects while examining the overall vitality of the tree. After that, a more thorough examination of the defects was conducted^25^. Considering the total number of trees in our study sample and the possibility of performing calculations, we applied four risk classes adapted from the TRAQ system^46^ and used VTA and ISA BMP methodology. The trees studied were classified into the following classes: low (A), moderate (B), high (C), and extreme (D)^49^. The decision as to which risk level to place a tree in depended on expert knowledge and the experience of the assessors^61^.

Each tree was examined using sonic tomography (SoT) at two heights—approximately 30 cm and 200 cm from ground level. A digital map of wood density (tomogram) of the stems of the living trees was created (See: “Appendix 2”). After entering additional data, the mechanical break strength of the tree was determined at the SoT level. The following thresholds for fracture strength were established: fracture strength (151% and above), moderate fracture strength (101–150%), and no fracture strength (0–100%). The tomography result may be unreliable due to damage and defects in the tree structure (fused logs, frost cracks, and included bark). Each test result was verified by visual inspection, and those with unreliable results were excluded from further investigation^61^.

### Research questions

The main research questions were: Are hollow street trees more susceptible to trunk failure? Do cavities in a trunk affect the visually assessed vitality of the trees? Are there tree species that pose a higher risk when hollow? Is SoT-measured decay a reliable indicator when checking the structural integrity of a tree?

### Statistical analysis of the data

The following statistical methods were used to analyse the data: Spearman rank correlation, Mann–Whitney U test, Kruskal–Wallis H test, Dunn multiple comparison test, pairwise comparison of proportions with Holm correction Fisher exact test, chi-square independence test, and weighted linear regression. Nonparametric tests were used because of the small sample size.

Tree vitality classes and TRAQ risk classes were formed for the statistical analyses. The risk classes were divided into three groups: A—low risk, B—moderate risk, C + D—high risk and extreme risk. There was a low sample of D group; therefore, to obtain more balanced data (A—136, B—113, C—59, D—9), we merged groups C and D to perform the Fisher test. Considering the potential of periods of high occupancy in our study area [depending on a variety of factors (i.e., time of day, day of week, weather, etc.)], those classes potentially pose the most severe consequences and share some similarities, being the two highest-risk classes. Tree vitality was divided into the following groups: 0 + 0/1—best vitality, 1 + /2—moderate vitality, and 2 + 2/3 + 3—weakest vitality. Analyses were performed using the programme R version 4.1.1^79^.

## Results

### Analysis for the complete data set—hollow-bearing tree versus the risk of trunk fracture and tree vitality

The first calculation was performed for the entire data set. The study used two visual tree classification methods: Roloff's and tree risk classification. The results of the two methods were correlated using the Spearman correlation coefficient of 0.46 and the chi-square test for independence (Table 2), with a *p* value of < 0.0001. Table 2 shows that risk class A (low) is mainly associated with the best tree vitality class on Roloff’s classification (0, 1 and 1/2). Only 4% of these trees are in stages 2–3. Class B (moderate risk) is most commonly observed (55% of class B trees) in trees with crown vitality between 1 and 2. Only 10% of high and extreme risk trees (C and D) were found among the trees with the best vitality (Roloff's classes 1 and 2).

**Table 2:** Relation between the visual methods of tree vitality and risk assessment.

*p* < 0.0001	Roloff*	0 + 0\1	1 + 1\2	2 + 2\3 + 3
Tree class **	A	68 (50%)	63 (46%)	5 (4%)
	B	29 (26%)	62 (55%)	22 (19%)
	C + D	7 (10%)	26 (38%)	35 (51%)

*p* value of the chi-square test of independence. Percentages are calculated independently for each data row.

*In order to perform the Fisher test, trees of classes 0 + 0\1, 1 + 1\2, and 2 + 2\3 + 3 were combined.

**In order to perform the Fisher test, trees of classes C + D were combined.

Risk classes division: A low-risk class group, B moderate risk class, C + D high and extreme risk class group. Tree vitality classes division: 0 + 0/1: best vitality, 1 + 1/2; moderate vitality, 2 + 2/3 + 3: the weakest vitality.

**Figure 3:**
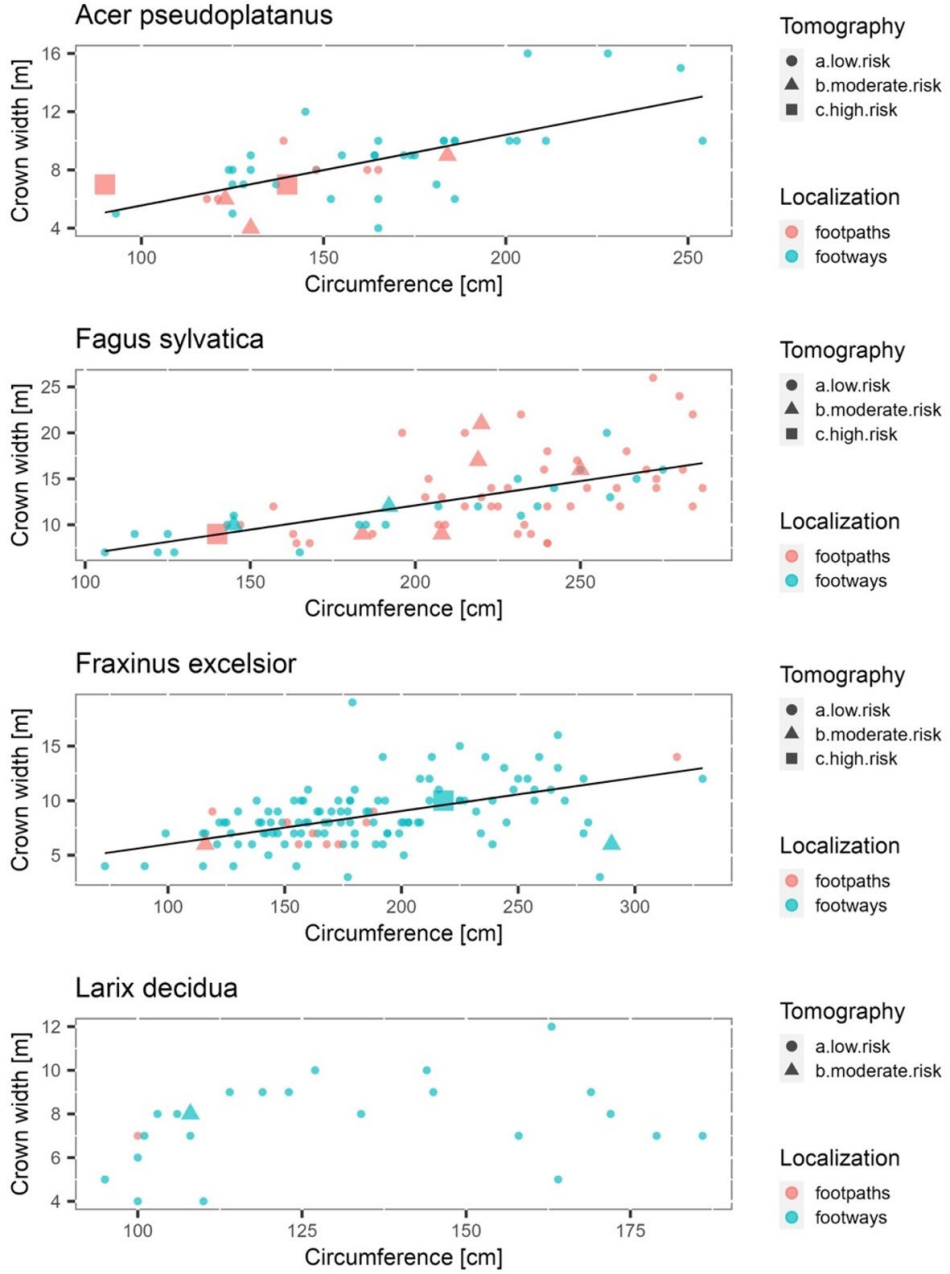
The relationship between tree size (crown width, circumference), tree location (footways along the streets, main footpaths in the park) and results from the tomography scan. The weighted regression line was added for each species. The line was not plotted for the *Larix decidua* because the dependence was non-significant. (*Source:* Processed by the Authors).

No statistically significant relationship was found between tree risk class/Roloff's class and tree hollows or SoT results; see Tables 3 and 4. The only indicator of tree vitality significantly related to the presence of cavities is sonic tomography (Table 4). Although the sample of trees that pose a moderate to high risk according to SoT is very small (17 and 5 trees, respectively), the majority (15 and 4, corresponding to 88% and 80%, respectively) were found to have cavities. It is worth noting that these trees represent only a small fraction of the 171 trees with visible hollows (2.3% and 8.8%, respectively). Finally, as seen in Fig. 3, the SoT results were unrelated to the tree risk classes and Roloff's classes.

**Table 3:** Relation between the visual methods of tree vitality and risk assessment.

	Hollow and/or fungi	
	Unobserved	Observed
Roloff * (*p* value = 0.27)		
0 + 0\1	42 (40%)	62 (60%)
1 + 1\2	71 (47%)	80 (53%)
2 + 2\3 + 3	33 (53%)	29 (47%)
Tree class ** (*p* value = 0.83)		
A	64 (47%)	72 (53%)
B	53 (47%)	60 (53%)
C + D	29 (43%)	39 (57%)
**Localization (*p* value = 0.0004)**		
Along the footways by a street	122 (52%)	112 (48%)
Along the footpaths in a park	24 (29%)	59 (71%)

*p* value of the chi-square test of independence. Percentages are calculated independently for each data row.

*In order to perform the Fisher test, trees of classes 0 + 0\1, 1 + 1\2, and 2 + 2\3 + 3 were combined.

**In order to perform the Fisher test, trees of classes C + D were combined.

**Table 4:** Characteristics of trees according to the sonic tomography analysis. Percentages are calculated independently for each data row.

	High risk	Moderate risk	Low risk
Tree status	5 (1.6%)	17 (5.4%)	295 (93.0%)
**Hollow and/or fungi (*p* value = 0.0032)**			
Unobserved	1 (0.7%)	2 (1.4%)	143 (97.9%)
Observed	4 (2.3%)	15 (8.8%)	152 (88.9%)
**Location (*p* value = 0.0018)**			
Along the footways by a street	2 (0.8%)	7 (3.0%)	225 (96.2%)
Along the footpaths in a park	3 (3.6%)	10 (12.0%)	70 (84.3%)
Roloff * (*p* value = 0.70)			
0 + 0\1	1 (1%)	6 (6%)	97 (93%)
1 + 1\2	4 (3%)	9 (6%)	138 (91%)
2 + 2\3 + 3	0 (0%)	2 (3%)	60 (97%)
Tree class ** (*p* value = 0.22)			
A	1 (1%)	4 (3%)	131 (96%)
B	2 (2%)	7 (6%)	104 (92%)
C + D	2 (3%)	6 (9%)	60 (88%)
**Tree species**			
*Abies concolor*	1	–	
*Acer platanoides*	–	1	
***Acer pseudoplatanus***	2	3	
*Aesculus hippocastanum*	–	1	
*Betula pendula*	–	1	
***Fagus sylvatica***	1	7	
***Fraxinus excelsior***	1	2	
***Larix decidua***	–	1	
*Tilia cordata*	–	1	

*p* values of the Fisher exact test. In bold: tree species with total samples exceeding 20, chosen for detailed analysis.

*In order to perform the Fisher test, trees of classes 0 + 0\1, 1 + 1\2, and 2 + 2\3 + 3 were combined.

**In order to perform the Fisher test, trees of classes A + B and C + D were combined.

Trees containing cavities or representing a moderate to high risk were more common along park paths than streets (Tables 3 and 4). A likelihood of the breakdown of trees that pose a high or moderate risk to the SoT by species can be found in Table 4.

### Analysis of the selected tree species

Data collection on trees in Zakopane was limited by the number of trees of each species growing in the study area. As shown in Table 1, most of the 23 species were represented by one to three specimens. A more detailed analysis of more than 20 samples was conducted across four species (*Acer pseudoplatanus* L., *Fagus sylvatica* L., *Fraxinus excelsior* L., *Larix decidua* Mill.). These species accounted for 83% of all trees studied. Their examples are illustrated in Fig. 2. The differences between the sample trees across the four species are shown in Table 5.

**Table 5:** Comparison of the characteristics of the examined trees of four species: *F. sylvatica*, *Fraxinus excelsior, Acer pseudoplatanus* and *Larix decidua*. Comparison of circumference, the width of tree crowns and optic tomography results using the Kruskal–Wallis H-test.

	Species	*Fagus sylvatica*		*Fraxinus excelsior*		*Acer pseudoplatanus*		*Larix decidua*	
	No. of trees	73		120		45		24	
Location of high and moderate-risk trees according to the optical tomography	Along the footpaths (in parks)	49 (67%)	1 high 5 moderate	10 (8%)	1 moderate	11 (24%)	2 high 3 moderate	1 (4%)	–
	Along the footways (by streets)	24 (33%)	2 moderate	110 (92%)	1 high 1 moderate	34 (76%)	–	23 (96%)	1 moderate
Tree circumference [cm]	Mean	216 a		186 b		161 c		130 d	
	Median	223		178		164		121	
	Stand. div	46.6		49.9		36.6		29.6	
Crown width [m]	Mean	13.0 a		8.6 b		8.5 b		7.6 b	
	Median	12		8		8		8	
	Stand. div	4.3		2.7		2.7		1.9	
Tomography	Mean	530 b		1241 a		742 b		502 b	
Share of trees at a moderate or high-risk rate		11.0%		2.5%		11.1%		4.2%	
Share of trees with hollows		74% a		45% b		29% b		42% b	
Roloff	0 + 0\1	43 (59%)		27 (23%)		9 (20%)		7 (29%)	
	1 + 1\2	24 (33%)		55 (46%)		32 (71%)		17 (71%)	
	2 + 2\3 + 3	6 (8%)		38 (32%)		4 (9%)		–	

According to Dunn's multiple comparisons test, species denoted by different letters differ in given characteristics. Comparison of proportions of trees with hollows and/or fungi performed with pairwise proportion test with Holm correction. The relationship between the species and Roloff’s classes was analysed using the chi-square test of independence. All presented relationships are significant at a *p* value < 0.0001.

The selected species differed in average circumference (measured at the height of 1.3 m) and crown width. According to the data distribution determined by Kruskal–Wallis H-test analysis, *F. sylvatica* had the largest trunk circumference and crown width. The other species did not differ in crown width in contrast with the distribution of their circumference. *F. excelsior* had a large average stem circumference, *A. pseudoplatanus* had a medium circumference, and *L. decidua* had a small circumference. Additionally, the four species differed in the distribution of their sites. *F. sylvatica* is the only species in which the majority (two-thirds) of its trees grow along the main paths in the selected city parks, while those from the other species grow mainly along the paths in the centre of Zakopane.

The vitality and cavities of the trees, which are the primary focus of this study, varied significantly in terms of characteristics amongst the selected species. As shown in Table 5, the samples of four tree species differed in terms of their condition estimated by Roloff’s classification. The most significant proportion of trees in the high condition (0 − 0/1) was found in *F. sylvatica* and the lowest in *F. excelsior* (2–3). Trees in moderate conditions dominated the samples of *A. pseudoplatanus* and *L. decidua* (1 − 1/2). Trees also differed numerically in the proportion of those classified as moderate or high risk on sonic tomography. Over 10% of these belonged to the species *F. sylvatica* and *A. pseudoplatanus*, and less than 5% belonged to the species *L. decidua* and *F. excelsior*. No relationship was found between species and risk classes.

Finally, the proportion of hollow trees varied amongst the four groups, with the highest proportion being found in *F. sylvatica* (74%). Across the other species, hollow trees accounted for up to 45%.

As with the full data analysis, no statistically significant relationships were found between the tree risk class or tree Roloff class and the occurrence of tree cavities or the results of tomographic analysis of tree trunks for the four individual tree species. The results of two assessments, classification into risk classes and Roloff’s classes, were correlated with each other for three of the four species studied: *F. sylvatica* (*p* value < 0.0001 for both Fisher’s exact test and Spearman’s correlation), *F. excelsior* (*p* value < 0.0001), and *A. pseudoplatanus* (*p* value < 0.05). Spearman correlation coefficient values ranged from 0.40 to 0.53.

Since only a minimal proportion of trees with moderate or high risk were observed to be indicated by sonic tomography, no statistically significant analysis of their characteristics can be performed for individual species. Nevertheless, the sizes (circumference vs crown width) of the higher-risk trees compared to the other trees are shown in Fig. 3. Weighted regression lines were added for each species to facilitate the interpretation of the data (ordinal linear regression was not used because of the heteroscedasticity of the data; no line was plotted for *L. decidua* because the relationship between tree circumferences was not significant). As can be seen, there does not appear to be a specific scheme for the size of the hazardous trees. They are not grouped according to circumference size or crown width. Similarly, no correlation was found between tree size and the occurrence of cavities in any of the tree samples.

Of the tree species studied, *F. sylvatica* is the most common along park footpaths (Table 4, Fig. 2). It is also the only species with a significantly higher proportion of hollowed trees in the parks (40 out of 49). On roadsides (13 out of 23), species exhibited hollows, confirmed by Fisher's test with *p* = 0.046. In *A. pseudoplatanus*, the percentage of trees with hollows is higher along the park footpaths (5 out of 11) than those in the streets (8 out of 34). However, the difference is not statistically significant. For *F. sylvatica*, trees in parks were larger on average than trees along roads. The opposite was observed for *A. pseudoplatanus* trees.

According to the Mann–Whitney U test, the differences between the mean circumferences and crown widths of the two examined species were significant at a *p* value of < 0.05. For *F. sylvatica*, trees in parks were larger on average than trees along roads. The opposite was observed for *A. pseudoplatanus* trees.

## Discussion

Our investigation focused on urban trees with tree trunk cavities that grow along pedestrian pathways and are subject to close monitoring by city officials, arising from public pressure driven by safety concerns versus other urban trees. Although not all trees along pavements in Zakopane were studied, due to the limited project budget, the study included trees in the most potentially high-risk locations. Additionally, due to the limitations of tomography, some of the trees studied could not be examined because the structure of the trunk did not provide a reliable diagnostic result.

The studied trees grow in a harsher habitat, alongside pedestrian walkways than other urban trees^80^. Crown architecture, which is considered a valuable indicator of crown condition and changes during the lifecycle of the tree^75^, may also be partially determined by site conditions^81,87^. The premature appearance of traits generally associated with age could be detected using Roloff’s classification^82,83^. Roloff's vitality phases 2 and 3 are typical of ageing trees, including premature senescence. Premature ageing- cavities and fungal fruiting bodies—may increase the tendency to remove trees from urban streetscapes^16^. The trees selected for the study can be considered mature and senescent, including prematurely aged trees, e.g., beech trees with trunk circumferences ranging from 100 to 287 cm or ash trees with trunk circumferences ranging from 90 to 329 cm. Because the above limitations lead to decisions on removing street trees for safety reasons being made too superficially, it was critical to verify whether cavities and/or fungal fruiting bodies posed an increased risk of trunk fractures.

23 species of trees were studied, with the majority having very few representatives. Therefore, the four most abundant species were selected for detailed analysis: *A. pseudoplatanus*, *F. sylvatica*, *F. excelsior* and *L. decidua.* These species varied in average size and were measured based on tree circumference and crown width. The trees also differed in average SoT scores as well as their propensity to be at moderate or high risk. The selected species also differed in vitality scores according to Roloff’s classification and the proportion of hollow trees.

The study results confirm that Roloff’s vitality and tree risk classes are closely related. Furthermore, the correlation between the results of the two approaches is consistent, irrespective of tree species. This result can be explained by the fact that dead branches can lead to a higher risk class, a typical sign, especially for older trees^21^ or those under habitat stress^80^. A tree would be classified in the high Roloff class in both cases.

On the other hand, no correlation was found between risks associated with stem breakage (SoT scan) and vigour. Those classified as hazardous by SoT may occur among trees of all Roloff or risk classes. It should be noted that the tomography results determine the likelihood of tree trunk failure. Therefore, they focus on the mechanical strength of the trunk (safety factor). Static and vitality are two different things—even a resistant tree can break. In a study by Terho and Hallaksela^67^, 14% of old, large urban trees that had fallen and posed a potential hazard had a vigorous and balanced crown and high recreational value^30^.

Unlike the SoT method, expertise can be used to examine the overall risk of trees. The risk of a tree falling or breaking can affect any part: the roots, root collar, trunk, crown collar, or crown^77^. Therefore, expertise is critical to the risk assessment decision-making process. According to Koeser and Smiley^61^, experienced professionals have lower risk ratings and are less likely to recommend tree removal as a risk mitigation measure. However, the fact that some trees that fall into the high-risk group according to SoT are classified by professionals as high (Roloff’s classes 0–1) shows an inability to determine the degree of loss of fracture strength of the stem by visual inspection and indicates the need for the use of such objective tools as SoT. The decision-making benefits of being supported by technical information^30^ are essential for both meeting the landowner's road safety obligation, as well as cutting urban trees that are valuable to urban residents based on subjective assumptions^16^.

Trees containing cavities accounted for more than half (54%) of all trees surveyed. However, only a small proportion was classified as hazardous by the SoT method, about 2% at the moderate risk level and 9% at the high-risk level. That being said since 19 of 22 (86%) hazardous trees were found to have cavities, it can be concluded that the occurrence of cavities increases the risk of trunk failure. However, since most hollow trees belong to the low-risk group, according to the SoT, the occurrence of cavities cannot be considered a leading indicator of the condition of the tree trunk. Therefore, it can only be used as an indication for applying the SoT or a similar method.

Some researchers confirm that one of the two most common types of tree failure is fractures i.e., decay and hollows that cause breaking branches and stems^28,30–32,44,84^. However, not only cavity presence but also the eccentricity of the damaged part impacts strength loss and tree stability^85^. Our study brings evidence that trunk hollows statistically do not have an adverse effect on tree vitality. Although previous research showed that improper maintenance, like heavy pruning, may result in exposure to fungal infection and a higher risk of failure in mature trees^36,37,86^, a cavity/hollow presence should not be perceived as a death sentence for the tree. The final decision on tree removal should be deeply considered and supported using SoT analysis.

The appearance of a cavity may indicate that the tree does not have enough healthy wood to remain standing. Nevertheless, most hollow trees are not dangerous^35,87,91^. Two facts can explain this. Firstly, the cavities found may differ depending on the stage of their development. Secondly, the abundant growth of wound wood on both sides of the cavity may compensate for the weakness caused by rot. This reaction wood is often stronger than normal stem wood^18,75,77^. Our results confirm previous studies: in general, trees with decayed stems pose low to moderate risk, and only a portion requires mitigation measures. However, hollow trees are at risk of branch failure and should be avoided through proper risk management^16,72^. Wessolly^72^ has found that many full-crown trees with trunk diameters greater than 1 m have only 5 to 10 cm wall thickness and yet have withstood all severe storms for decades^72^. We found one of the highest results, 3869%, of stem strength in *F. excelsior*. Tree No. 51 has a 66 cm stem diameter (DBH) and is 24% hollow (See: “Appendices 1, 2”). Our results show that hollow mature and ageing trees less than 1 m in diameter confirm trunk fracture strength. Tests, especially SoT, can confirm the risk level, but this issue needs expect cavities to be more common in trees in higher-risk classes. Wolf found cavities were more further investigation.

One might common in trees with low vigour and visible signs of woodpecker predation than in trees with high vigour^88^. In contrast, the present research indicates that the presence of a tree cavity does not correlate with poor tree vigour^88,92^. Zajączkowska et al. proves that even almost hollow tree may stand for over 700 years and provide efficient photosynthetic capacity^92^. None of the risk class ratings of the trees studied correlated with the presence of cavities. Analysis of the individual tree species showed that while cavities were most frequently observed in *F. sylvatica*, trees of this species were also most frequently in the best condition, according to Roloff’s classification.

One might also expect that trees with cavities and a high risk of trunk breakage would be associated with the largest trees since tree size should indicate tree life expectancy. However, the present study found no such association for individual species. Nevertheless, a significant difference in the frequency of cavity occurrence was found amongst the four selected species. Furthermore, there were many more hollow trees in the *F. sylvatica* sample compared to the others. This increase in numbers may be related to the average size of *F. sylvatica* trees, both in stem and crown width, which exceed the trees from the other three species.

Unfortunately, the sampling of each species was not balanced due to natural constraints in their location: park trails versus roads (Table 1). The only two species that exceeded 20% in parks were *F. sylvatica* (67%) and *A. pseudoplatanus* (24%). In both cases, the proportion of trees with cavities was higher in parks than in streets. Since the tree samples from the above two species contained mostly park trees, the excess of hollow trees along the footpaths in parks was observed in all data.

We have focused on the possible risk that may pose hollow/cavity or fungi presence in relation to tree vitality and trunk fracture. The issue of target occupancy and risk rates in relation to the likelihood of impact could be studied deeply as the next step in our research.

In addition, high or moderate-risk trees were found more frequently in the park than along the streets. The higher proportion of hollow ash and beech trees along park footpaths could be due to managers' lower risk acceptance. This could result in the felling of trees with cavities growing along roads being considered hazardous, resulting in a loss of biodiversity^16,18^. These results appear to oppose the results of Klein et al.^28^, indicating that arborists more often perceive trees as a danger to pedestrians' safety rather than to vehicular traffic. Interestingly, when asked to rate consequences of failure for various diameter branches, most arborists selected “significant” to “severe” for stems equal to or greater than 29.2 cm when the target was a pedestrian and for vehicular targets, this threshold was 69.2 cm^65^. Therefore, technical inspection should be included in the sustainable management process to complement cavity tree monitoring*.*

Furthermore, ash trees in our study were statistically distinguished from the species studied by poorer vitality (high Roloff's classes) but not by high-risk class. Within each, there was a statistically significant difference in the breaking strength of the trunk with almost 50% hollow trees (mean 1242%). The weakened vigour can be explained by the response to difficult site conditions as a species with low-stress tolerance^3,22,23^. Because urban roadway conditions are characterised by compacted soils, elevated pH, and heavy metal concentrations^80^, it is likely that tree roots near roadways are also damaged. Hightshoe^89^ and Costello and Jones^90^ consider ash to be reasonably tolerant of root loss. Read^93^ refers to its intermediate tolerance or even intolerance to pruning. In addition, the vigour of European ash may be weakened by ash dieback. Infested trees seem more susceptible to other secondary pathogens, such as *Armillaria ssp.*, especially in unfavourable urban conditions^62,63^. In our study, the factor of poor site conditions could be the cause of the weakened condition of one of the studied species.

Based on the available material, we conclude that tree risk assessment expertise is critical when deciding on urban tree removal. However, only tomography-based methods can provide reliable data on tree risk because hollows, cavities and fruiting bodies are not indicative of risk classes in most cases. We found that the size of trees in urban environments does not affect the number of hollow trees (in general or within individual species) but that only a small number of trees with cavities or fungal fruiting bodies pose a risk.

More detailed studies of the hollowness and decay of different tree species are needed. Our next goal is to analyse the effects of street tree root system health with respect to cavities and fruiting bodies. Root damage associated with the installation of pathways and pavements can lead to decay upslope and increase the risk of trunk damage, especially at the lower levels. Therefore, it is essential to determine whether poor root system condition is associated with hollows/cavities and rot in the lower portions of street tree trunks translates into trees becoming higher risk as determined by tomography.

## Supplementary Information

Supplementary Information 1.

Supplementary Information 2.

## Data Availability

The datasets generated during and/or analysed during the current study are available from the corresponding author upon reasonable request.
