# Supplementary material for: Potential hazard characteristics of trees with hollows, cavities and fruiting bodies growing along pedestrian routes
Source: Sci Rep. 2022 Dec 10;12:21417. doi: 10.1038/s41598-022-25946-0 (PMC9741646; doi:10.1038/s41598-022-25946-0)
Supplement: Supplementary file 1 — Supplementary Information 1. [file 41598_2022_25946_MOESM1_ESM.docx]

# **Potential hazard characteristics of trees with hollows, cavities and fruiting bodies, growing along pedestrian routes**

**Measurement report Arborsonic 3D**

Tree no 51

*04.07.2021 18:08*

**Tree species:** *Fraxinus excelsior*

| **Tree location** | Kościuszki Street |
| --- | --- |
| **Measurement date** | 14/06/2021 time: 11:09 |
| **Tree ID** | 51(82SG) |
| **DBH** | 66 |

Biomechanics

| **Tree crown** | |
| --- | --- |
| Crown area | 27.4 m^2^ |
| Distance from trunk collar to: | |
| Top of the crown | 9.61 m |
| Middle of the crown | 6.33 m |
| **Tree trunk** | |
| Degree of inclination | 89 ° |
| **Wind** | |
| Wind speed | 33,0 m/s |
| Wind load | 3806 N |
| Resistance factor | 0,2 |
| Compressive strength of the stem | 26 MPa |

| **Layer** | **Height of measurement** | **The extent of tree cavity inside the trunk section** | **Safety factor** | **Risk level** |
| --- | --- | --- | --- | --- |
| Layer 1 | 200 cm | 1 % | 4001 % | Low risk |
| Layer 2 | 30 cm | 24 % | 3869 % | Low risk |

**Safety factor:** 3869 %

**Layer 1**

Geometry of the sensors

| Height | 200 cm |
| --- | --- |
| Scheme | Circle |
| No. of sensors | 10 |

Sensor positions

| C | 210 |
| --- | --- |
| PD | 3 |
| BT | 2 |

**Warstwa 2**

Geometry of the sensors

| Height | 30 cm |
| --- | --- |
| Scheme | Circle |
| No. Of sensors | 10 |

Sensors positions

| C | 230 |
| --- | --- |
| PD | 3 |
| BT | 2 |


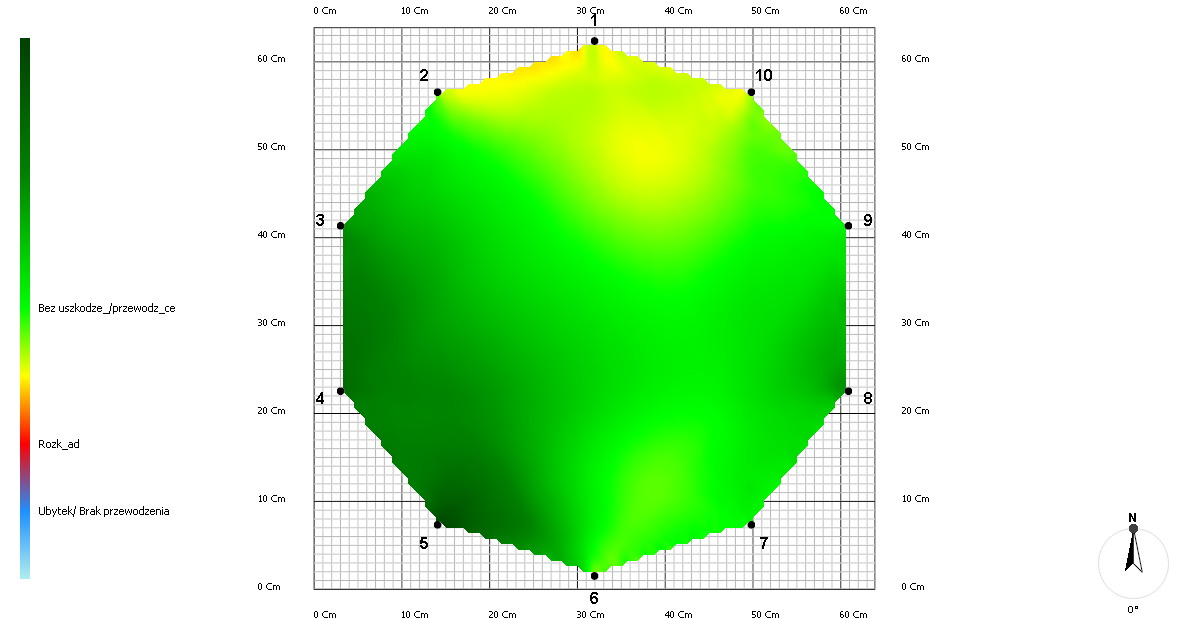


*Layer 1*


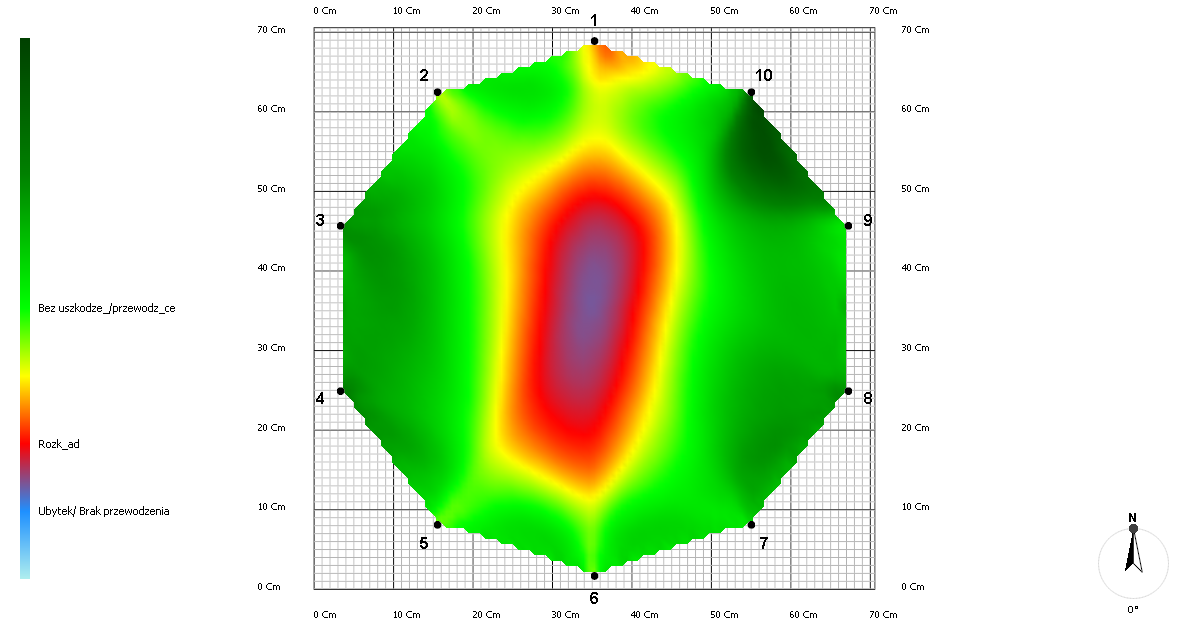


*Layer 2*
