# Supplementary material for: Potential hazard characteristics of trees with hollows, cavities and fruiting bodies growing along pedestrian routes
Source: Sci Rep. 2022 Dec 10;12:21417. doi: 10.1038/s41598-022-25946-0 (PMC9741646; doi:10.1038/s41598-022-25946-0)
Supplement: Supplementary file 2 — Supplementary Information 2. [file 41598_2022_25946_MOESM2_ESM.docx]

**Supplementary material 1.**

**Sonic tomograms with tree image for selected trees.**

1. solid wood percentage in the tomogram image 0% (left), visual tree appearance of tree and the trunk (right),

Kościuszki Street, No 18(32SG)


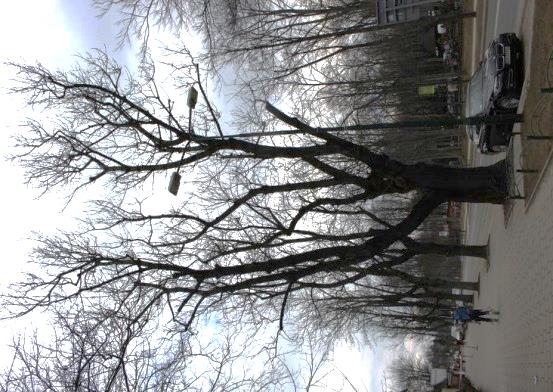

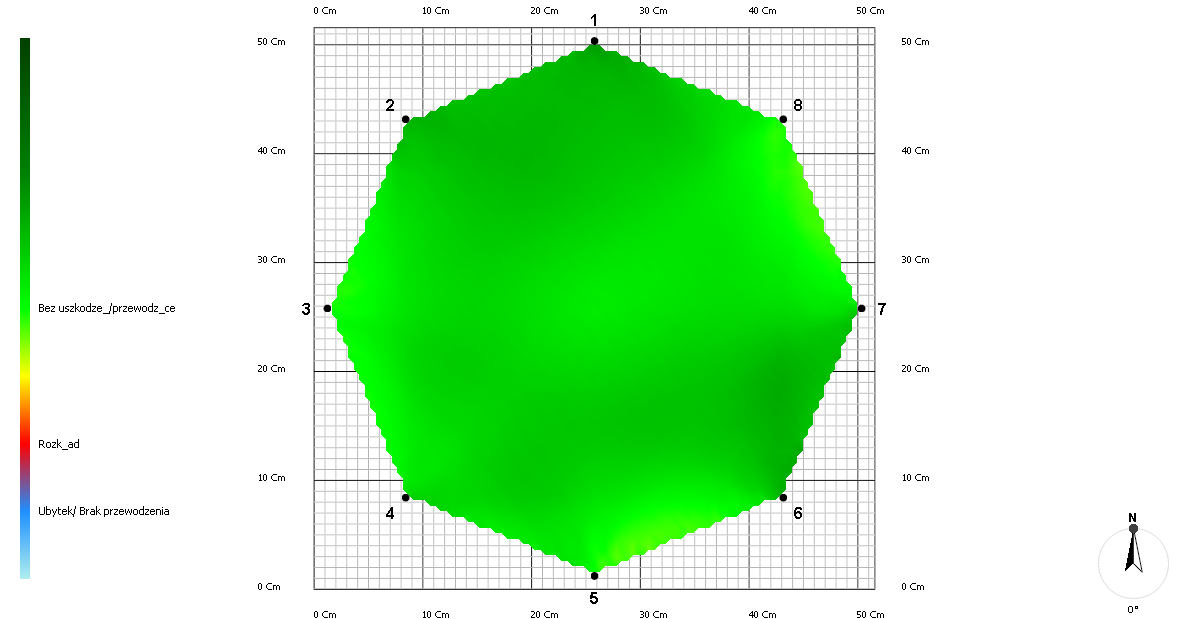


*Layer 1*


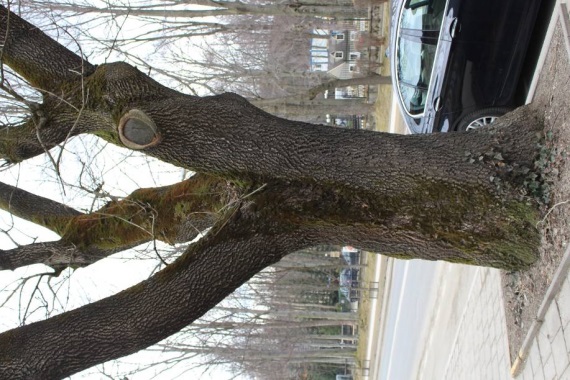

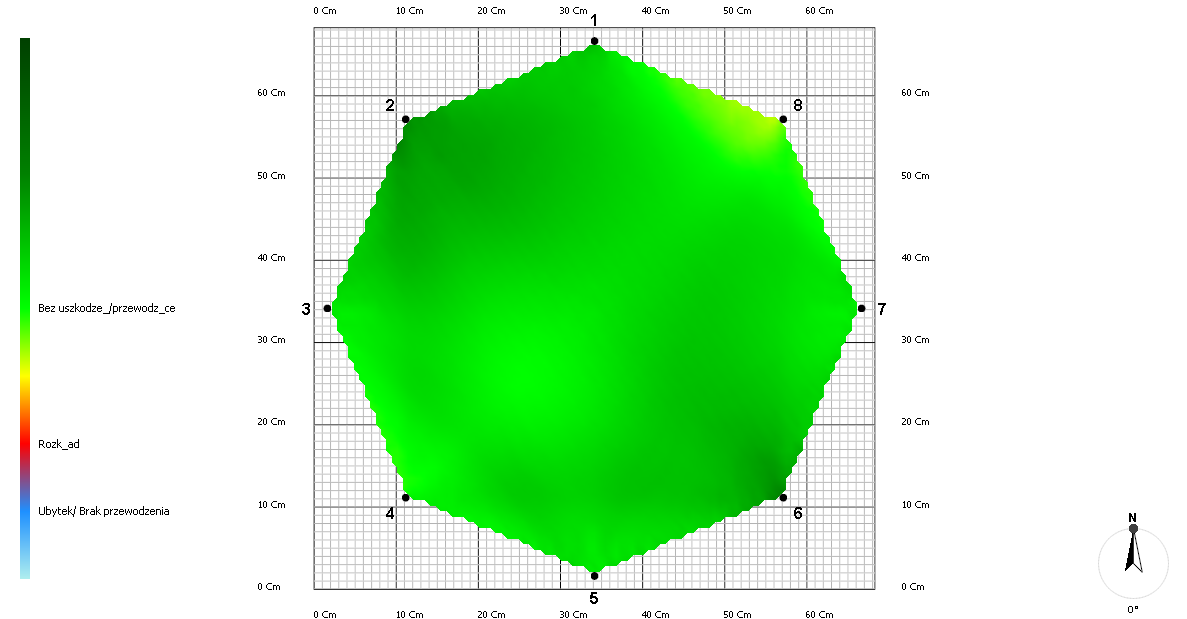


*Layer 2*

| **Layer** | **Height of measurement** | **The extent of tree cavity inside the trunk section** | **Safety factor** | **Likelihood of stem fracture** |
| --- | --- | --- | --- | --- |
| Layer 1 | 200 cm | 0 % | 1560 % | Low risk |
| Layer 2 | 30 cm | 0 % | 2449 % | Low risk |

1. hollow trunk base (40% of cavity) and solid wood percentage on the trunk layer (0%) in the tomogram image (left), visual tree appearance (right),

Kościuszki Street, No 38(69SG)


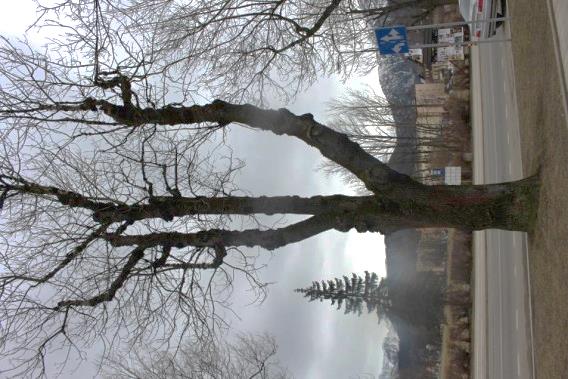

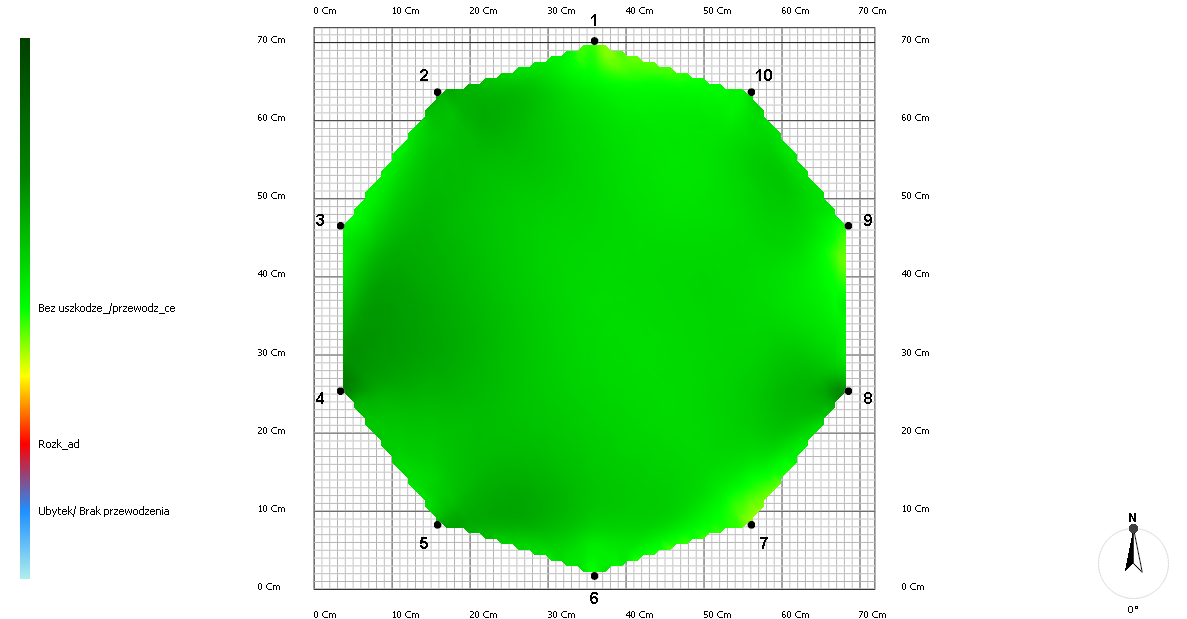


*Layer 1*


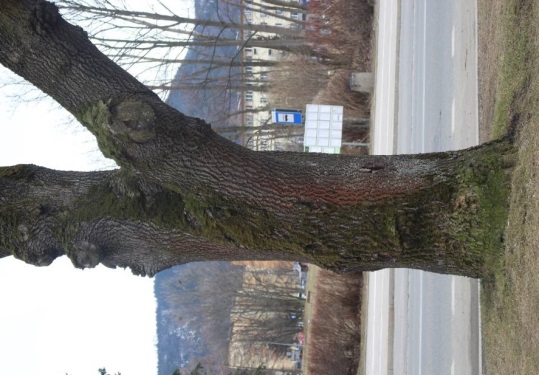
*
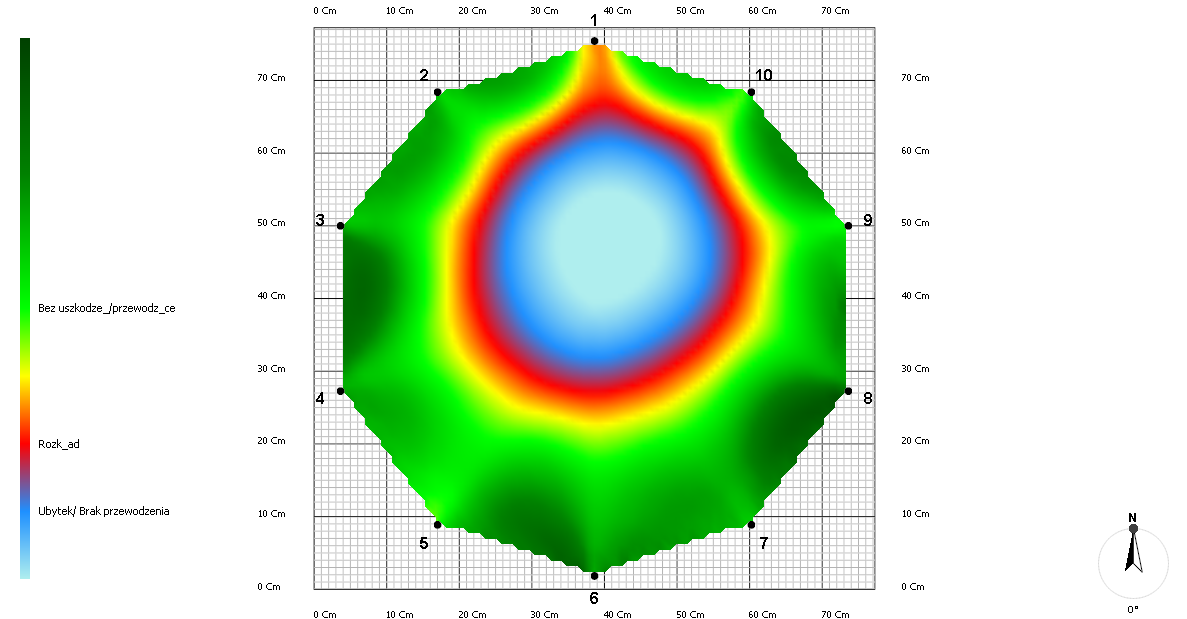
*

*Layer 2*

| **Layer** | **Height of measurement** | **Tree cavity area inside the trunk section** | **Safety factor** | **Likelihood of stem fracture** |
| --- | --- | --- | --- | --- |
| Layer 1 | 200 cm | 0 % | 2108 % | Low risk |
| Layer 2 | 30 cm | 40 % | 1904 % | Low risk |

1. hollow trunk base (50% of cavity) and cavity presence on the trunk layer (25%) in the tomogram image (left), visual tree appearance (right)

Ulica Kościuszki 34(61SG)


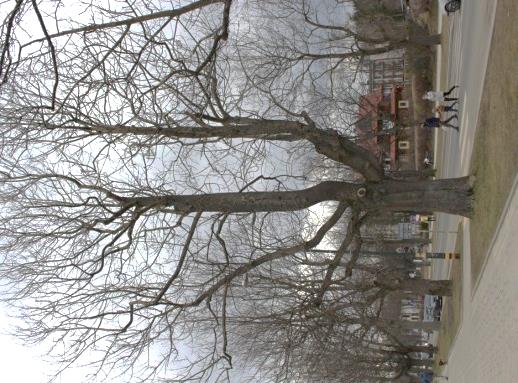

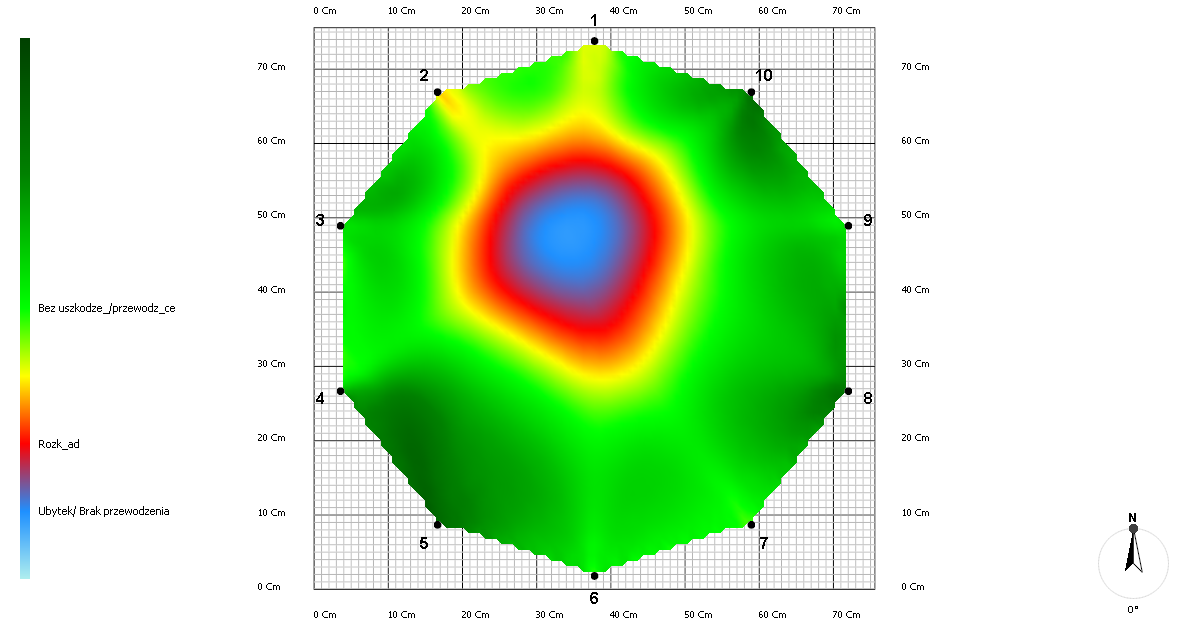


*Layer 1*


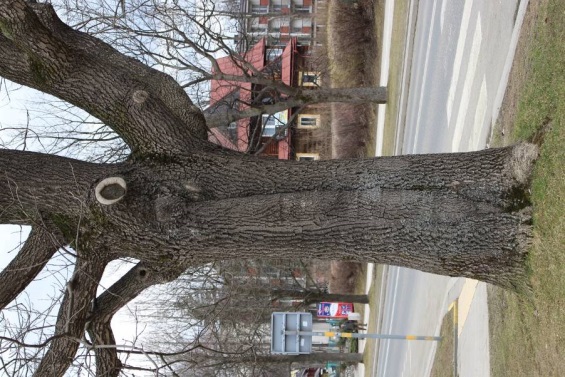
*
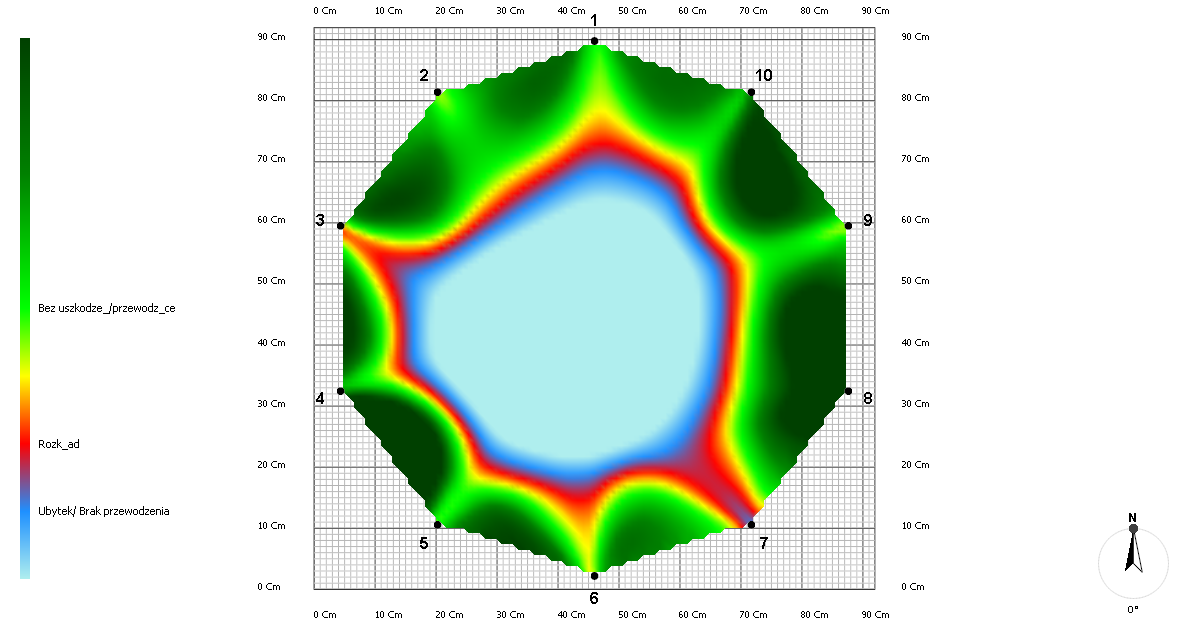
*

*Layer 2*

| **Layer** | **Height of measurement** | **Tree cavity area inside the trunk section** | **Safety factor** | **Likelihood of stem fracture** |
| --- | --- | --- | --- | --- |
| Layer 1 | 200 cm | 22 % | 1155 % | Low risk |
| Layer 2 | 30 cm | 50 % | 1215 % | Low risk |
